# Silica hydrogels as a carbon-free solid media for the culture of diverse organisms

**DOI:** 10.1093/femsmc/xtae035

**Published:** 2024-12-28

**Authors:** Druhi Vaid, Alisa Zubir, Alistair Hanak, Tanda Qi, Daniela Delneri, Lu Shin Wong

**Affiliations:** Manchester Institute of Biotechnology, University of Manchester, 131 Princess Street, Manchester M1 7DN, United Kingdom; Department of Chemistry, University of Manchester, Oxford Road, Manchester M13 9PL, United Kingdom; Manchester Institute of Biotechnology, University of Manchester, 131 Princess Street, Manchester M1 7DN, United Kingdom; Department of Chemistry, University of Manchester, Oxford Road, Manchester M13 9PL, United Kingdom; Manchester Institute of Biotechnology, University of Manchester, 131 Princess Street, Manchester M1 7DN, United Kingdom; Faculty of Biology, Medicine and Health, University of Manchester, Oxford Road, Manchester M13 9PT, United Kingdom; Manchester Institute of Biotechnology, University of Manchester, 131 Princess Street, Manchester M1 7DN, United Kingdom; Faculty of Biology, Medicine and Health, University of Manchester, Oxford Road, Manchester M13 9PT, United Kingdom; Manchester Institute of Biotechnology, University of Manchester, 131 Princess Street, Manchester M1 7DN, United Kingdom; Faculty of Biology, Medicine and Health, University of Manchester, Oxford Road, Manchester M13 9PT, United Kingdom; Manchester Institute of Biotechnology, University of Manchester, 131 Princess Street, Manchester M1 7DN, United Kingdom; Department of Chemistry, University of Manchester, Oxford Road, Manchester M13 9PL, United Kingdom

**Keywords:** tetraethoxysilane, inorganic gel, hydrogel, biocompatible, solid culture media, selective culture media

## Abstract

Bacteriological agar plates are commonly used to carry out experiments for the selective growth of microorganisms and the isolation of single-strain colonies. However, the presence of agar itself may be a confounding factor since it may serve as a source of carbon and energy. Moreover, there have been ongoing constraints on the production and sourcing of agar. These concerns have led to an interest in the development of agar substitutes. Silica hydrogels are entirely inorganic carbon-free polymeric materials that lack any source of micronutrients. Herein, a revised method for the preparation of silica hydrogels as a solid culture medium is reported. These gels can be formulated with a range of nutrient-rich or minimal media supplemented with various carbon sources, and can be manipulated in the same manner as agar gels. Their use for the culture and isolation of diverse microorganisms, including both Gram-positive and Gram-negative bacteria, yeast, and filamentous fungi is demonstrated. These silica hydrogels supplemented with either antibiotics or other molecules of interest can also be used for microbial selection experiments.

## Teaser

Silica hydrogel solid culture media as a carbon-free alternative to agar.

## Introduction

Bacteriological-grade agar was first reported as a solid culturing media for microorganisms in 1882 and has since become a staple of microbiological research because of its convenient handling characteristics including high gel strength, low gelling temperature, non-toxicity, stability over a wide range of temperatures, and ease of preparation (Armisén 1991, Armisén and Gaiatas 2009). These properties have led to the widespread use of agar, instead of other gelling agents such as guar gum, alginates, or starch.

At a molecular level, agar is a hydrogel composed of a network of agarose, a polysaccharide derived from red seaweed (*Gelidium* or *Gracilaria* sp.). *Gelidium* populations give higher yields of agar with lower gelling temperatures compared to agar derived from *Gracilaria*, making the latter less suitable for bacteriological and technical applications (Porse and Rudolph 2017). However, since *Gelidium* cannot be readily cultivated, the ever-increasing demand of agar has resulted in overharvesting, with consequent effects on their habitat, and fluctuations in the supply and cost of agar (Callaway 2015, Santos and Melo 2018).

Agar plates that are produced with nutrient media (e.g. LB broth) are routinely used to carry out experiments involving the selective growth of microorganisms and the isolation of single-strain colonies. For example, where a compound is used to inhibit the growth of all except the microorganism of interest (e.g. antibiotics), or where a nutrient of interest is added to determine if a particular microorganism is capable of utilizing it for growth. However, in the latter case, the presence of agar itself may be a confounding factor since it may serve as a source of carbon and energy (Payton et al. 1976). In this context, alternative solid media materials that are free of carbon sources would be desirable.

Silica hydrogels are entirely inorganic gels consisting of a loose network of polymeric silicon oxide with trapped water, and in isolation do not contain any carbon, nitrogen, phosphorous, or other micronutrients. These hydrogels can be synthesized via the ‘sol-gel’ process from a variety of small molecule precursors, including inorganic silicates such as sodium silicate, or alkoxysilanes such as tetraethyl orthosilicate (TEOS) or tetramethyl orthosilicate (Homburg and Patel 2022). Gels with a range of mechanical and physicochemical properties can be produced, depending on their synthesis conditions (i.e. type and ratios of precursors and gelation catalyst, aging time, and temperature) (Pietras-Ożga et al. 2022).

In microbiology, silica gels have been applied in a variety of experiments, including the encapsulation of microbes within the silicate matrix, with subsequent applications as biosensors or bioreactors (Brányik et al. 1998, Coradin and Livage 2007, Sakkos et al. 2017, Li et al. 2023). Various formulation of silica hydrogels in streak plate format for microbiological purposes has been known since at least 1949 (Hutton and ZoBell 1949, Temple 1949, Kingsbury and Barghoorn 1954, Funk and Krulwich 1964, Dietz and Yayanos 1978, Bazylinski and Rosenberg 1980). These gel preparations were later used in environmental microbiology experiments to exclude the effect of any agarolytic activity (Ohhata et al. 2007). However, previous efforts to produce silica gels using inorganic silicates have suffered from poor control of their mechanical properties (i.e. gel stiffness) which made them difficult to handle in practical microbiology (Coradin and Livage 2007, Baccile et al. 2009). Furthermore, it has been found that these gels were difficult to use because of their watery consistency that resulted in the diffusion of the colonies (i.e. not possible to isolate individual ones) and any attempts at drying them resulted in gel cracking (Ohhata et al. 2007). Due to the limitations of the existing silica hydrogels and the ongoing risk of agar supply disruptions, the formulation of these silica gels was revisited with the aim of developing materials that can be conveniently used for routine microbiological applications. Herein, a method to produce silica hydrogels using TEOS is reported. These gels can be used in streak plate format to culture a range of microorganisms, including both Gram-positive and Gram-negative bacteria, yeast, and filamentous fungi. Moreover, the use of these silica hydrogels for selection experiments is demonstrated wherein this solid medium can be supplemented with compounds to selectively grow only microbes of interest.

## Materials and methods

### Materials

*Escherichia coli* BL21 (DE3) competent cells (referred to henceforth simply as *E. coli*) were purchased from New England BioLabs (Ipswich, MA, USA). Where necessary, *E. coli* was transformed with pET-28a vector using the protocol provided by the supplier with only minor modifications (see SI). *Bacillus subtilis* 168, *Pseudomonas putida* KT2440, and *Marinobacter nauticus* VT8 were purchased from DSMZ-German Collection of Microorganisms and Cell Cultures GmbH (Braunschweig, Germany) as freeze-dried microorganisms and were revived according to the instructions provided by the supplier. *Saccharomyces cerevisiae* BY4743 was purchased from the European Saccharomyces Cerevisiae Archive for Functional Analysis (EUROSCARF, Oberursel, Germany). *Saccharomyces cerevisiae* isolate 96.2 was provided courtesy of Eladio Barrio (Arroyo-López et al. 2010). *Aspergillus Fumigatus* A1160 was purchased from the Fungal Genetics Stock Centre (Manhattan, KS, USA). TEOS was purchased from Fisher Scientific UK Ltd (Loughborough, UK) and used without further purification.

### Preparation of silica hydrogels

TEOS (17. 4 ml) was vigorously mixed with ethanol (11.2 ml) and distilled water (11.2 ml, adjusted to a pH of 2.8 using 6 N HCl), and the solution allowed to stand at 4°C for 2 days. To a 7 ml aliquot of this solution was then added 1 N NaOH (70 μl). To produce the streak plates, either 4.5 ml or 6.5 ml of this TEOS/NaOH solution was mixed immediately with either 14.5 ml nutrient rich media or 13.5 ml minimal media supplemented with substrate of interest (Table 1), respectively; then poured into glass Petri dishes. The poured mixtures were left to stand for 30 min during which gelation occurred. The gels were covered with 10 ml water and subsequently incubated at 62°C overnight to evaporate any residual ethanol. Subsequently, any excess water was decanted prior to use.

**Table 1.** List of microorganisms cultured on silica hydrogels with the corresponding media and incubation temperature used.

Microorganism	Media recipe	Incubation temperature
*E. coli* BL21	Luria–Bertani (LB) media, containing (in g l^−1^): tryptone (10.0), NaCl (10.0), yeast extract (5.0)	37°C
*E. coli* BL21 with pET-28a plasmid	LB media supplemented with 20 μl of 50 mg ml^–1^ kanamycin	37°C
*B. subtilis* 168	LB media	30°C
*P. putida* KT2440	LB media	30°C
*S. cerevisiae* BY4743	Yeast-Peptone-Dextrose (YPD) media, containing (in g l^−1^): peptone (20.0), dextrose (20.0), yeast extract (10.0)	30°C
*S. cerevisiae* 96.2	Yeast Nitrogen Base (YNB) media containing (in g l^−1^): Yeast-Nitrogen-Base without amino acids and without ammonium sulphate (1.9), ammonium sulphate (5.0)	30°C
*A. fumigatus* A1160	Sabouraud-Dextrose Broth (SDB) media, containing (in g l^−1^): dextrose (20.0), pancreatic digest of casein (5.0), peptic digest of animal tissue (5.0)	37°C
*M. nauticus* VT8	Halomonas Complex Media (HCM), containing (in g l^−1^): casamino acids (7.5), yeast extract (1.0), NaCl (50.0), MgSO_4_ (11.0), sodium citrate (3.0), K_2_HPO_4_ (0.5), FeSO_4_(NH_4_)_2_SO_4_ (0.05)	30°C
*E. coli* BL21 vs. *P. putida* KT2440	M9 media (M9), containing (in g l^−1^): Na_2_HPO_4_·7H_2_O (12.8), KH_2_PO_4_ (3.0), NaCl (0.5), NH_4_Cl (1.0), MgSO_4_ (0.24), CaCl_2_ (0.01) Gels had a final concentration of 1.0% *n-*butanol	30°C
*E. coli BL21* vs. *M. nauticus* VT8	M9 media (925 ml) supplemented with 75 ml of 20% w/v xylose Gels had a final concentration of 1.0% xylose	30°C

### Microbial culturing

For the bacteria and yeast, liquid cultures (20 ml) were grown overnight in the media appropriate to the organism under investigation (Table 1). After incubation, the cultures were pelleted by centrifugation and washed twice with water to remove any residual media. The cell pellets were re-suspended in ~200 μl of water and used for streaking on to the gels. For *A. fumigatus*, a loop of spores was gently scraped from a pre-cultured solid plate and streaked onto the gels. These inoculations were carried out in technical triplicate. Microbial growth was compared to negative control plates that were not inoculated.

## Results and discussion

### Gel formulation

The mechanical and physiochemical properties of silica hydrogels is largely dependent on synthesis parameters. Hence, to prepare silica hydrogels that possessed the physical properties that were similar to agar, parameters such as the ratio of reaction precursors, concentration of catalyst, as well as aging and drying temperatures were first established. In general, the gels were produced by pre-mixing the gelation components (TEOS, ethanol, acidified water), aging for a pre-determined period of time, followed by the addition of NaOH (to catalyse gelation) and either nutrient or minimal media.

Firstly, the ratios of TEOS, acidified water, and ethanol were investigated; and these mixtures were incubated at 4°C for periods of up to 2 days to test differences in gel properties such as gel consistency as well as gelation time (Brányik et al. 1998). It was found that a ratio of 3:2:2 (TEOS:acidified water:ethanol) and a 2-day incubation period at 4°C resulted in gels that were firm and could be streaked without breaking the surface. Increasing the amount of ethanol resulted in a material with a hard glass-like consistency whilst increasing the amount of water resulted in gels that were too soft and fragile, with streaking often resulting in breakage of the gel surface. Further, it was found that aging the mixtures for 2 days prior to gelation led to faster gel formation upon the addition of NaOH (under 30 min), with shorter aging resulting in mixtures that took extremely long periods of time to solidify (>5 days when aged for 1 day or if aging was omitted). The concentration of NaOH was also varied, and it was found that the gelation time decreased as the concentration of NaOH was increased, but >12.5 mM glass-like materials were formed. It was also observed that addition of NaOH not only decreased the gelation time, but it also adjusted the pH to ∼7, making the gels conducive to microbial growth.

**Figure 1.**
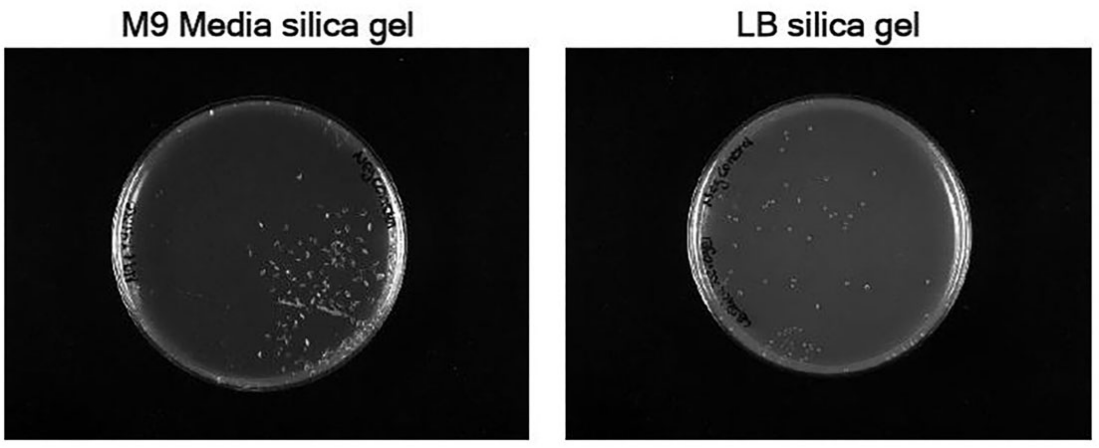
Images of silica gel plates before inoculation showing the presence of air bubbles within the gel.

Once the synthesis parameters were established, these gels were made with a range of media, wherein the TEOS/water/ethanol solution with NaOH was mixed with a liquid growth medium. In all cases, it was found that gels of an acceptable consistency were generated that could be streaked in the usual manner. Ethanol is produced as a byproduct of TEOS hydrolysis during the gelation process, but was seen as beneficial since it meant that the initial preparation of the gels could be carried out without aseptic conditions. However, to avoid any detrimental effects on microbial growth in the subsequent culture experiments, a heat treatment step was incorporated to drive off the ethanol prior to use. Here, it was found that incubation at 62°C overnight gave the best results. Shorter drying periods or lower temperatures did not evaporate all the ethanol from the gel (as evidenced by a lack of *E. coli* growth after inoculation on gels incorporating LB medium, results not shown). The heat treatment sometimes resulted in the formation of air bubbles within the gel (Fig. 1), but their presence did not break the surface and did not hinder microbial growth.

### Microbial inoculation and culture

The streaking of inoculum on to these plates was carried out in the same manner as with conventional agar gels. To demonstrate the general applicability of this solid medium, a range of microbes were cultured on silica gels containing the appropriate nutrient media (Table 2). These organisms included both prokaryotes (Gram positive and negative) and eukaryotes (yeast and filamentous fungi).

**Table 2.** List of microorganisms cultured on silica hydrogels with the corresponding nutrient medium.

Microorganism	Nutrient supplemented in gel	Comment
*E. coli* BL21	LB	Gram negative
*P. putida* KT2440	LB	Gram negative
*B. subtilis* 168	LB	Gram positive
*M. nauticus* VT8	HCM	Gram negative, halophile
*S. cerevisiae* BY4743	YPD	Yeast
*A. fumigatus* A1160	SDB	Filamentous fungi

**Figure 2.**
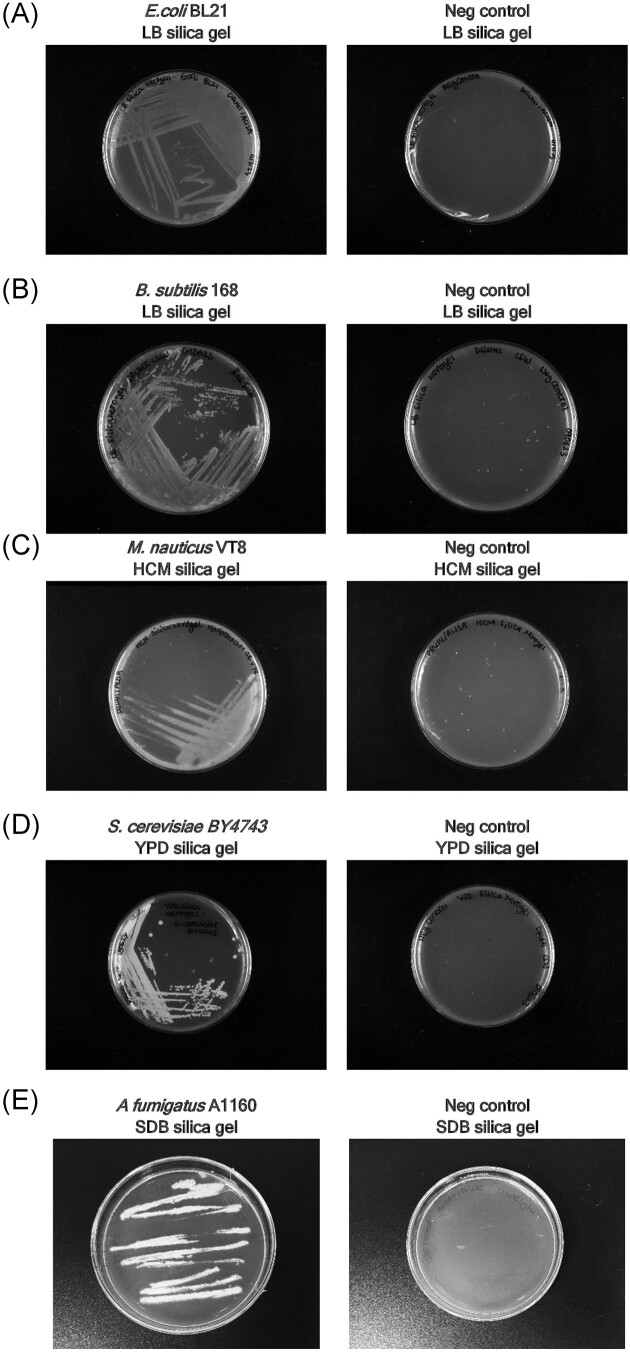
Images of silica gel plates: (A) *E. coli* grown on LB-supplemented gels; (B) *B. subtilis* grown on LB-supplemented gels; (C) *M. nauticus* grown on HCM-supplemented gels; (D) *S. cerevisiae* grown on YPD-supplemented gels; (E) *A. fumigatus* grown on SDB-supplemented gels. All growth experiments are shown next to corresponding negative controls (gels without inoculum). Images of triplicate experiments are shown in SI.

In all cases, these microorganisms were shown to successfully grow on silica gels (Fig. 2A–E, Figs S1–S5 in SI). The incubation times for the appearance of colonies were similar to those grown on agar plates, typically overnight for bacteria and under 2 days for yeast and filamentous fungi. The colonies obtained on these silica gels could be re-inoculated into fresh media and cultured, indicating that the cells remained viable when grown on these gels.

In order to test whether these silica hydrogels had any residual ethanol that could serve as a viable source of carbon, two further experiments were carried out whereby gels were prepared that did not contain any additional carbon source. Here, gels were prepared containing either M9 minimal medium and inoculated with *P. putida* KT2440; or containing YNB without amino acids and inoculated with *S. cerevisiae* 96.2, a strain that lacks nutritional deficiencies that inhibit growth in the absence of amino acids. In both cases, no colonies were observed after overnight growth (Fig. S6 in SI).

### Selective culture of microbes

**Figure 3.**
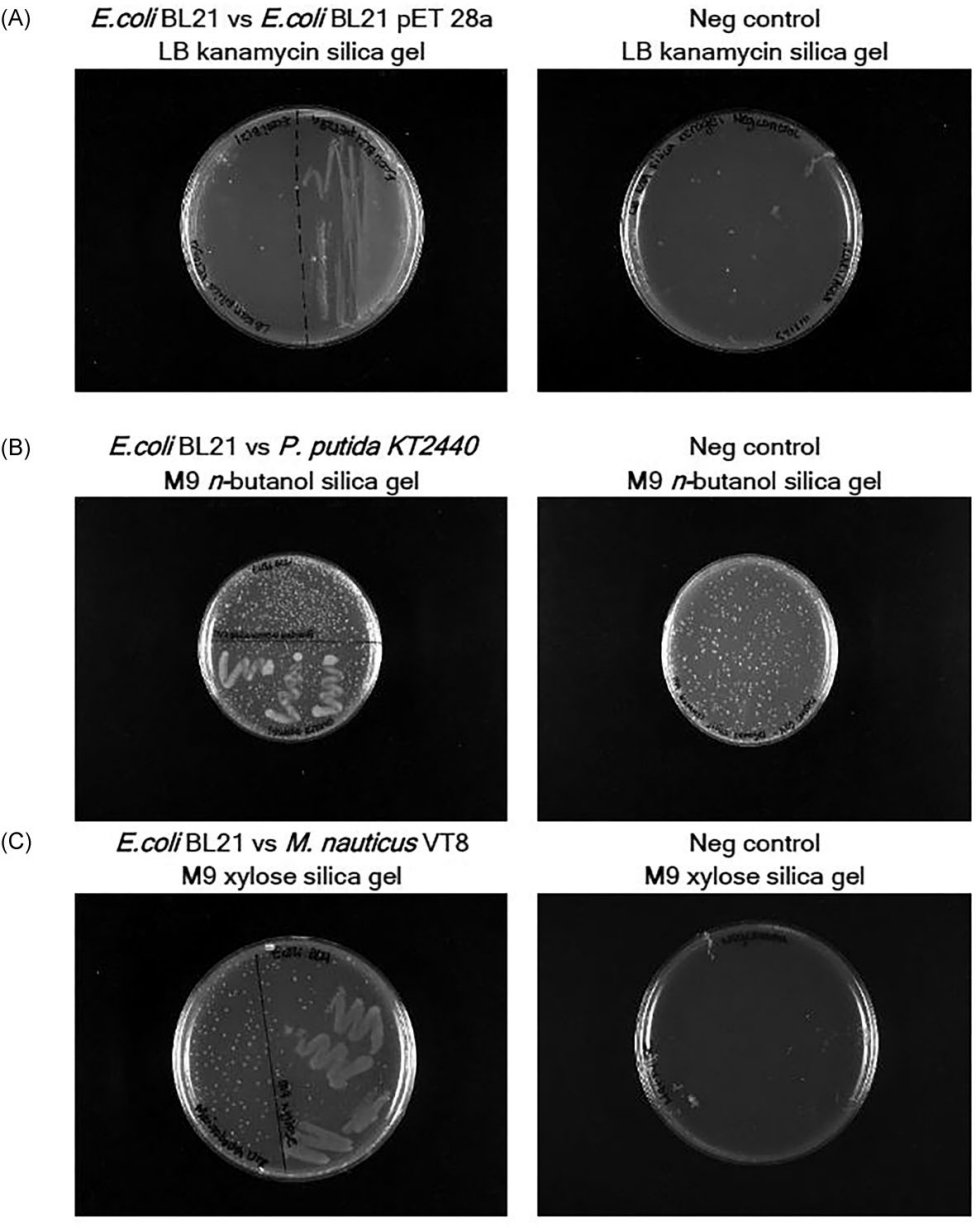
Images of selective microbial culture on silica hydrogels: (A) *E. coli* BL21 vs. *E. coli* BL21 pET-28a grown on kanamycin-containing gels; (B) *E. coli* BL21 vs. *P. putida* KT2440 grown on silica gels with M9 minimal media and *n*-butanol; (C) *E. coli* BL21 vs. *M. nauticus* VT8 grown on gels containing M9 minimal media and xylose. All growth experiments had corresponding negative controls (gels without inoculum), within which the presence of air bubbles formed during gel synthesis can be seen. Images of triplicate experiments are shown in SI.

As noted above, solid culture media are widely used to investigate the selective growth of microbes on media containing, or lacking, the compounds of interest. To demonstrate selection in the presence of an otherwise toxic compound, gels with LB media supplemented with kanamycin were streaked with *E. coli* BL21 that had been transformed with a pET-28a plasmid conferring kanamycin resistance, and compared with cells that were not transformed. It was found that selective growth could be obtained on these gels, with only the portions of the media inoculated with the resistant bacteria giving rise to colonies (Fig. 3A, Fig. S7 in SI).

To demonstrate the selection of microbes that are able to assimilate specific compounds, experiments were conducted whereby only one carbon source was provided. In the first example, hydrogels were synthesized with minimal media supplemented with 1% *n*-butanol. These gels were then inoculated with *P. putida* KT2440, which can utilize this alcohol as a sole source of carbon, or *E. coli* BL21 that cannot do so. Growth was readily observed on the regions of the plate inoculated with *P. putida* KT2440, but not with *E. coli* BL21 even after a 7-day incubation (Fig. 3B, Fig. S8 in SI). In the second example, the selective culture with xylose as the sole carbon source for *E. coli* BL21 was compared with *M. nauticus* VT8 (Liu et al. 2018). Here, only the *E. coli*-inoculated portions of the gel exhibited growth (Fig. 3C, Fig. S9 in SI).

## Conclusions

Silica hydrogels for use as solid culture media were successfully synthesized and their use as a carbon-free substitute to conventional agar plates was demonstrated. It was shown that these gels, when supplemented with the appropriate nutrients, can be used to culture and isolate a variety of microorganisms, including both Gram-positive and Gram-negative bacteria, yeast, and filamentous fungi. Furthermore, they can also be applied in various selection experiments. In principle, the method of formulating the gels reported here could also be applied to a range of parallelized formats (e.g. microtitre plates, chambered microscopy slides, etc.).

It is anticipated that these gels could serve as an alternative to agar in the event of supply shortages. As a carbon-free medium, it may also find applications in experiments in nutrient assimilation where the presence of the agar itself was a confounding factor. The nutrient versatility offered by silica hydrogels makes them a potential biotechnological solution for the isolation of organisms capable of biodegradation of various anthropogenic compounds.

## Supplementary Material

xtae035_Supplemental_Files

## Data Availability

The images generated during the current study are available in the Supplementary Information.
