# Supplementary material for: Silica hydrogels as a carbon-free solid media for the culture of diverse organisms
Source: FEMS Microbes. 2024 Dec 28;6:xtae035. doi: 10.1093/femsmc/xtae035 (PMC11737323; doi:10.1093/femsmc/xtae035)
Supplement: xtae035_Supplemental_Files [file xtae035_supplemental_files.zip › FEMSMC-2024-041.R1 one sentence summary.docx]

Silica hydrogel solid culture media as a carbon-free alternative to agar.
