# Supplementary material for: Silica hydrogels as a carbon-free solid media for the culture of diverse organisms
Source: FEMS Microbes. 2024 Dec 28;6:xtae035. doi: 10.1093/femsmc/xtae035 (PMC11737323; doi:10.1093/femsmc/xtae035)
Supplement: xtae035_Supplemental_Files [file xtae035_supplemental_files.zip › L58_SI_v6.docx]

**­­Supporting Information**

**Silica hydrogels as a carbon-free solid media for the culture of diverse organisms**

Druhi Vaid,^a,b^ Alisa Zubir,^a,b^ Alistair Hanak,^a,c^ Tanda Qi,^a,c^ Daniela Delneri,^a,c^ Lu Shin Wong^a,b*^

^a^ Manchester Institute of Biotechnology, University of Manchester, 131 Princess Street, Manchester M1 7DN, United Kingdom

^b^ Department of Chemistry, University of Manchester, Oxford Road, Manchester M13 9PL, United Kingdom

^c^ Faculty of Biology, Medicine and Health, University of Manchester, Oxford Road, Manchester M13 9PT, United Kingdom

* email: [l.s.wong@manchester.ac.uk](mailto:l.s.wong@manchester.ac.uk)

**Transformation protocol**

**Figure S1.** *E.coli* BL21 grown overnight on LB-supplemented gels and their corresponding negative controls (gels without inoculum)

**Figure S2.** *B. subtilis* 168 grown overnight on LB-supplemented gels and their corresponding negative controls (gels without inoculum)

**Figure S3.** *M. nauticus* VT8 grown overnight on HCM*-*supplemented gels and their corresponding negative controls (gels without inoculum)

**Figure S4.** *S. cerevisiae* BY4743 grown on YPD*-*supplemented gels and their corresponding negative controls (gels without inoculum)

**Figure S5.** *A. fumigatus* A1160 grown overnight on SBD*-*supplemented gels and their corresponding negative controls (gels without inoculum)

**Figure S6. (A)** *P. putida* KT2440 grown on M9 gel; **(B)** *S. cerevisiae* 96.2 grown on YNB gel. Both experiments are shown next to corresponding negative controls (gels without inoculum)

**Figure S7.** *E.coli* BL21 vs. *E.coli* BL21 pET-28a grown on kanamycin-containing gels and their corresponding negative controls (gels without inoculum)

**Figure S8.** *E.coli* BL21 *vs P.* putida KT2440 grown on M9 *n-*butanol supplemented gels and their corresponding negative controls (gels without inoculum)

**Figure S9.** *E.coli* BL21 *vs M. nauticus* VT8 grown on M9 xylose supplemented gels and their corresponding negative controls (gels without inoculum)

**Transformation protocol**

The supplier’s transformation protocol for BL21 (DE3) competent cells was used with some modifications. Briefly, one tube (50 µL) of BL21(DE3) competent *E. coli* cells were thawed on ice for 10 min and a 1.7 µL solution of ~100 ng of pET-28a plasmid DNA was added to the cell mixture. The tube was flicked carefully 4–5 times to mix cells and DNA. The mixture was placed on ice for 1 hr following which it was immersed into a heat block at 42 °C for 1 min. The mixture was subsequently placed on ice for 5 min. 900 µL of room temperature SOC was pipetted into the mixture which was then placed at 37 °C for 1 hr and shaken. The cells were sedimented by centrifugation (400 g, 5 min). 800 µL SOC was discarded and the cells were resuspended in the remaining solution, which was spread onto an agar-kanamycin selection plate and incubated overnight at 37 °C to obtain colonies.

| 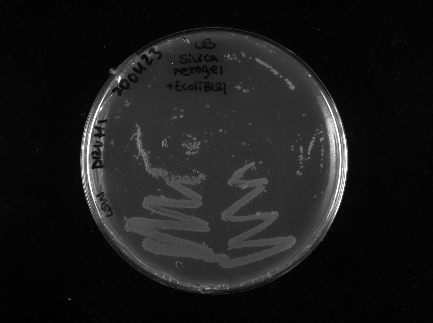 | 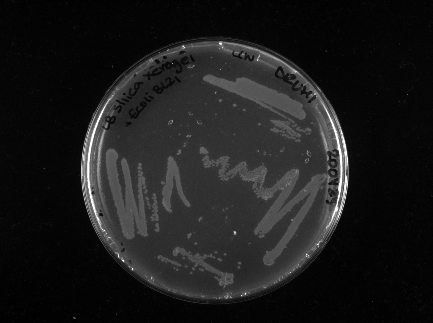  ***E.coli* BL21 LB silica gels** | 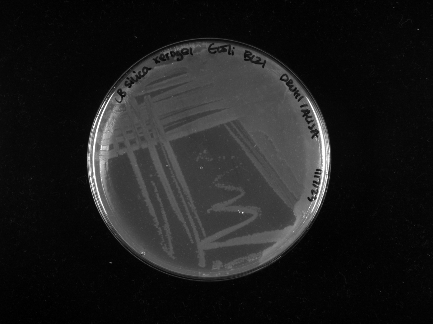 |
| --- | --- | --- |
| 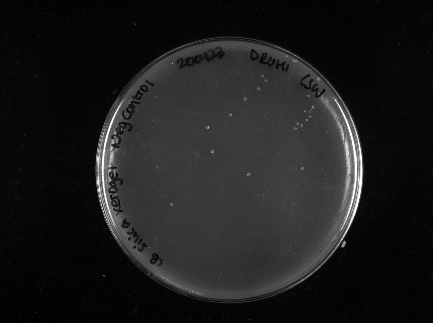 | 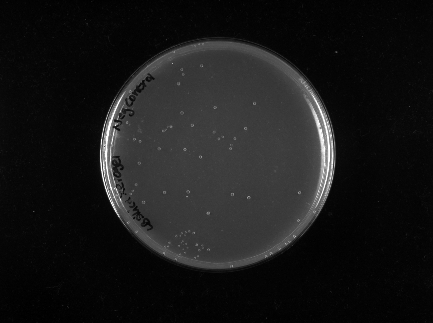  **Neg control - LB silica gels** | 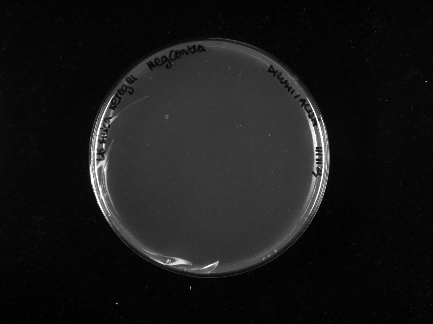 |

**Figure S1.** *E.coli* BL21 grown overnight on LB-supplemented gels and their corresponding negative controls (gels without inoculum).

| 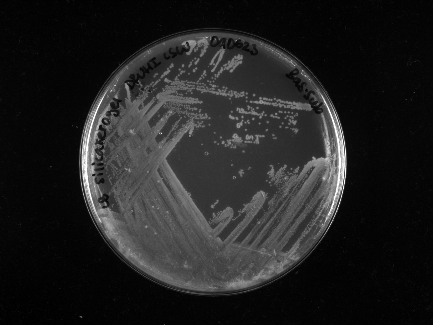 | 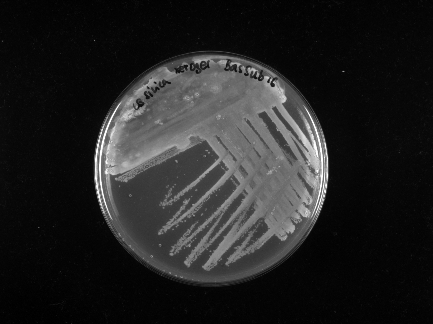  ***B. subtilis* 168 LB silica gels** | 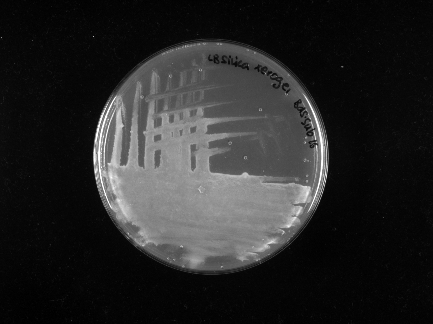 |
| --- | --- | --- |
| 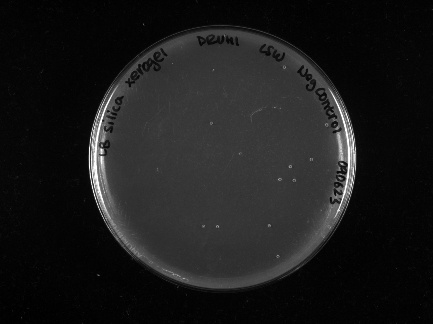 | 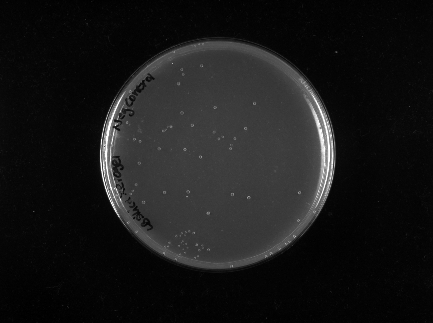  **Neg control - LB silica gels** | 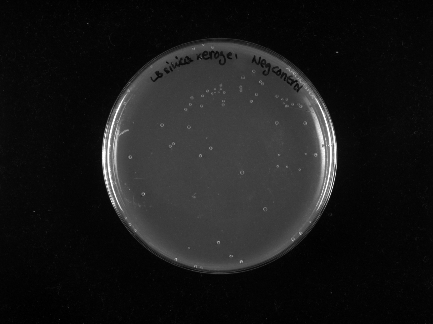 |

**Figure S2.** *B. subtilis* 168 grown overnight on LB-supplemented gels and their corresponding negative controls (gels without inoculum).

| 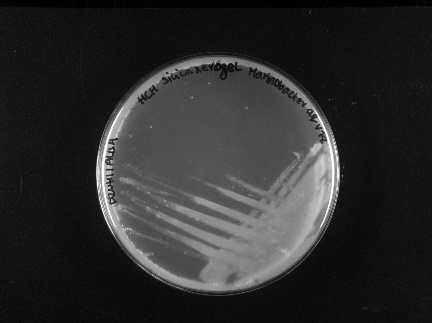 | 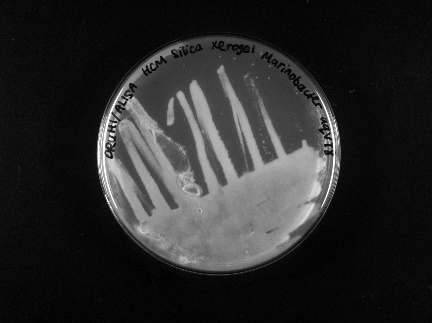  ***M. nauticus* VT8**  **HCM silica gels** |
| --- | --- |
| 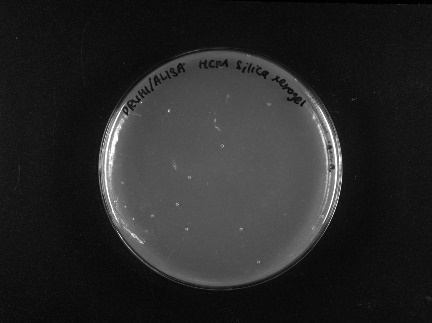 | 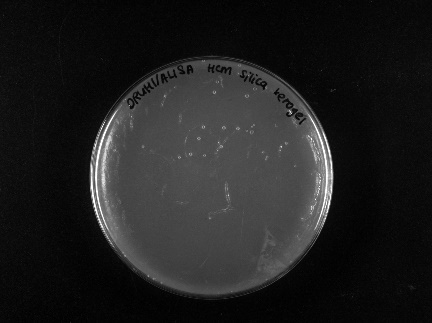  **Neg control - HCM silica gels** |

**Figure S3.** *M. nauticus* VT8 grown overnight on HCM-supplemented gels and their corresponding negative controls (gels without inoculum).

| 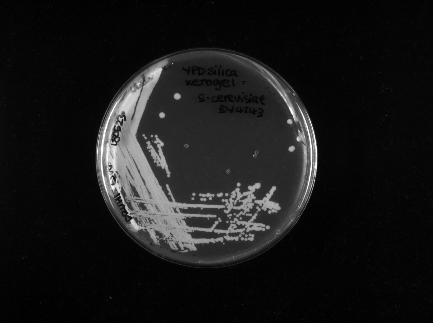 | 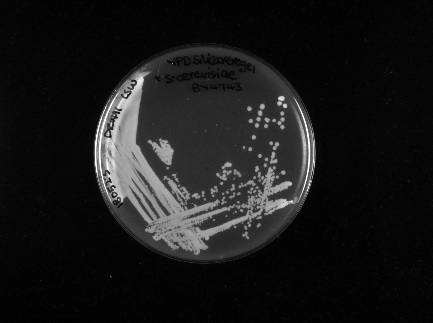  ***S. cerevisiae BY4743***  **YPD silica gels** | 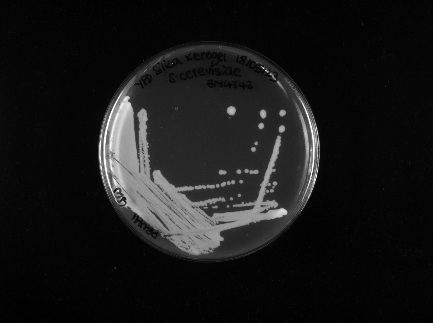 |
| --- | --- | --- |
| 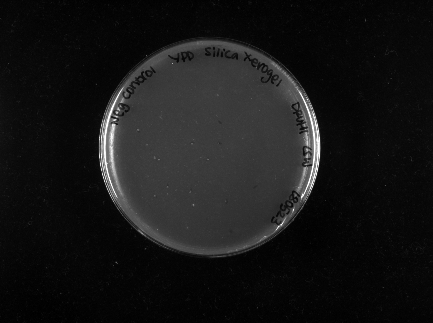 | 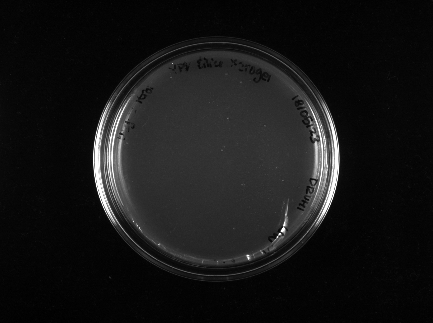  **Neg control - YPD silica gels** | 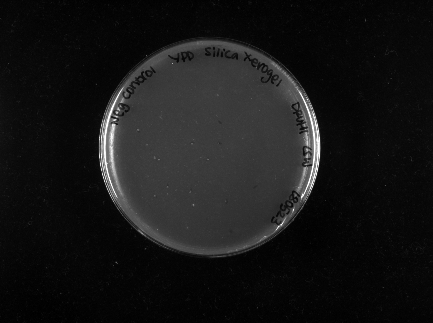 |

**Figure S4.** *S. cerevisiae* BY4743 grown on YPD-supplemented gels and their corresponding negative controls (gels without inoculum).

| 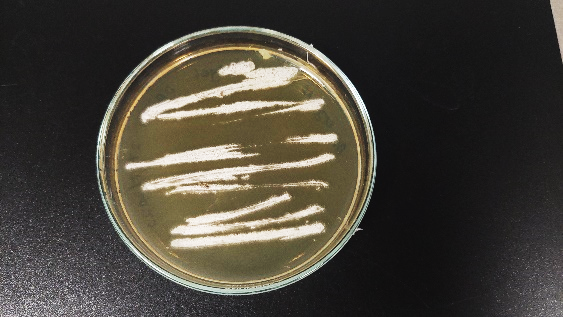 | 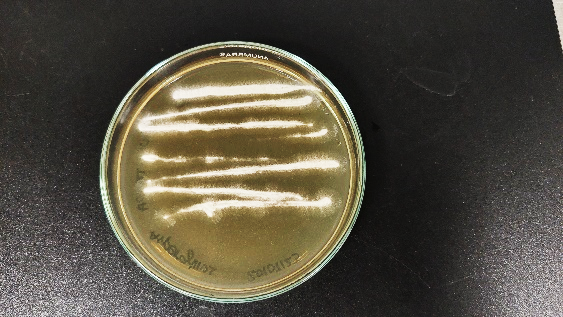  ***A fumigatus* A1160**  **SDB silica gels** | 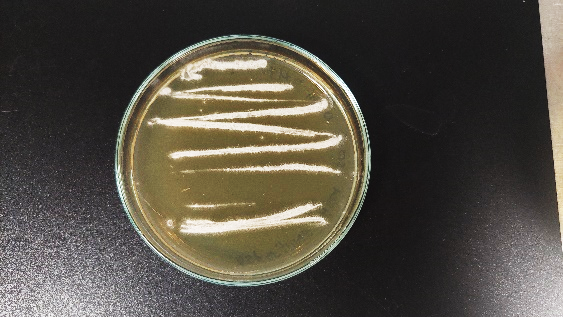 |
| --- | --- | --- |
| 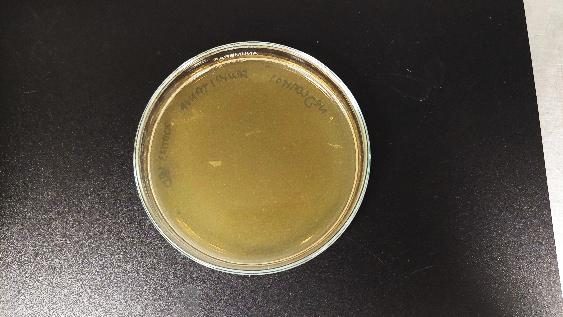 | 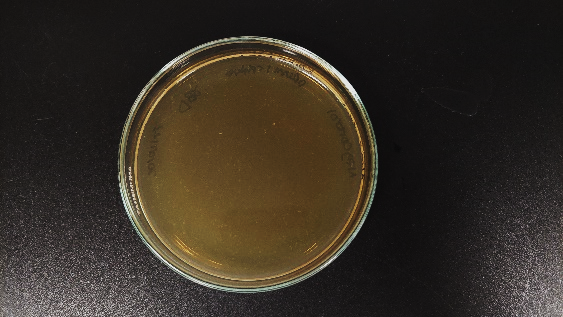  **Neg control - SDB silica gels** | 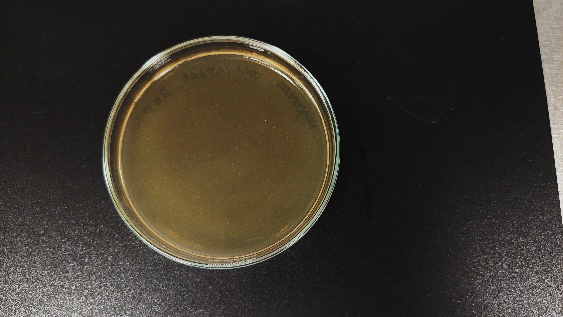 |

**Figure S5.** *A. fumigatus* A1160 grown overnight on SDB-supplemented gels and their corresponding negative controls (gels without inoculum).

| (A) | 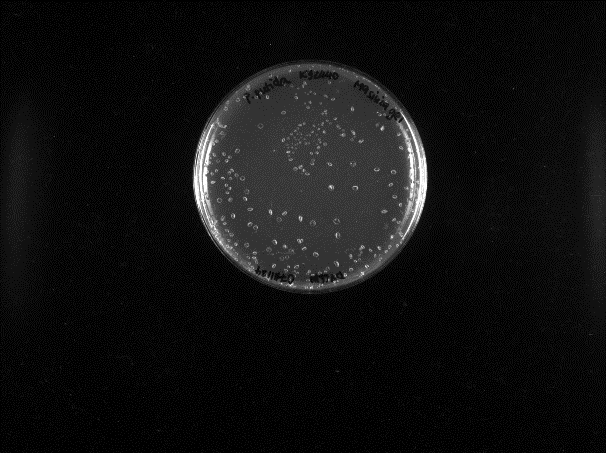  ***P. putida* KT2440**  **M9 silica gel**  ***Iynb SILICA GEL*** | 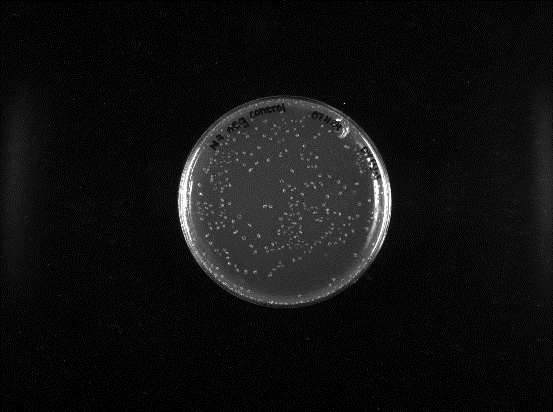  **Neg control**  **M9 silica gel** |
| --- | --- | --- |
| (B) | 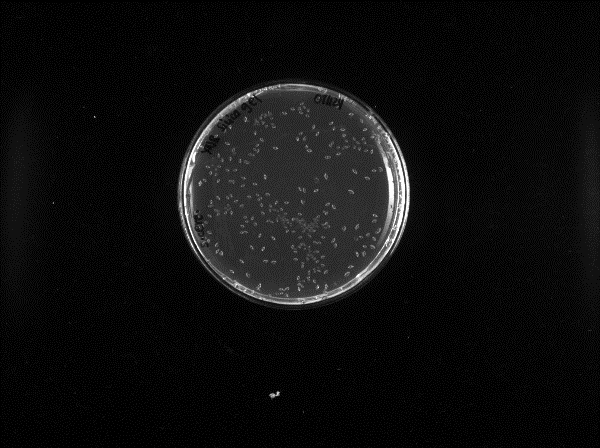  ***S. cerevisiae* 96.2**  **YNB silica gel**  ***Iynb SILICA GEL*** | 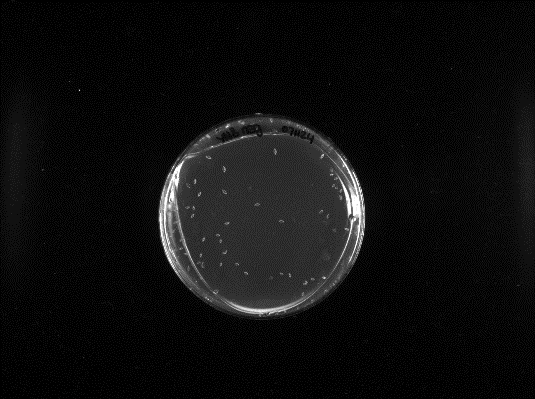  **Neg control**  **YNB silica gel** |

**Figure S6. (A)** *P. putida* KT2440 grown on M9 gel; **(B)** *S. cerevisiae* 96.2 grown on YNB gel. Both experiments are shown next to corresponding negative controls (gels without inoculum).

| 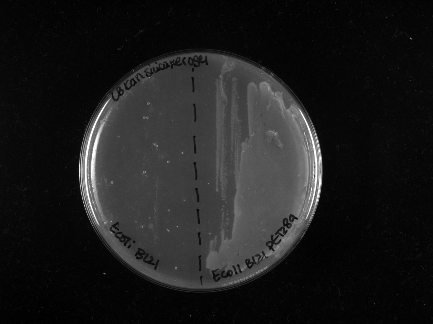 | 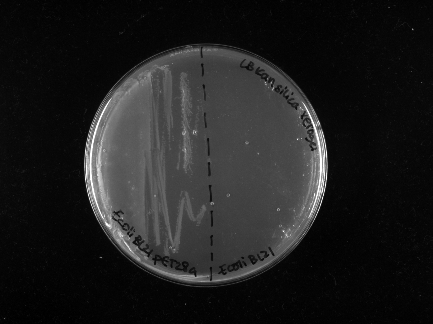  ***E.coli* BL21 pET 28a (left side of plate) vs *E.coli* BL21 (right side of plate)**  **LB kanamycin silica gels** | 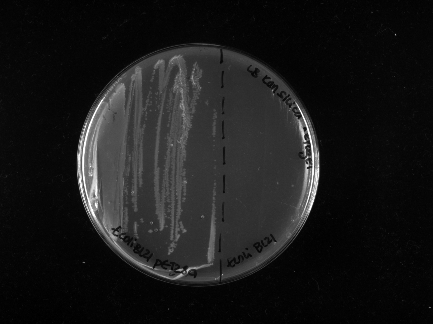 |
| --- | --- | --- |
| 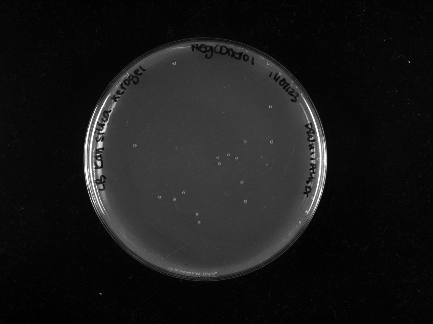 | 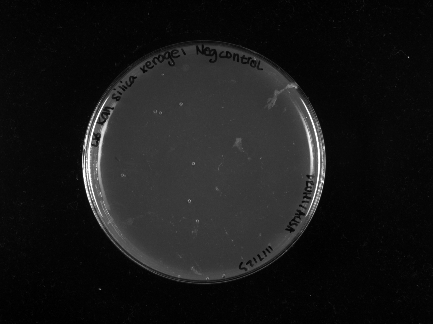  **Neg control – LB kanamycin silica gels** | 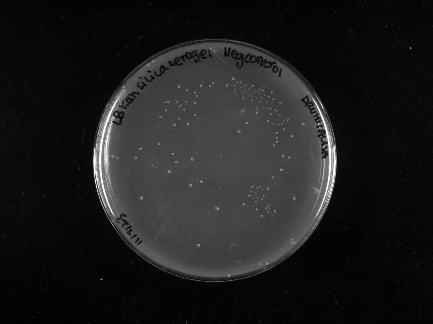 |

**Figure S7.** *E.coli* BL21 *vs E.coli* BL21 pET-28a grown overnight on kanamycin-containing gels and their corresponding negative controls (gels without inoculum).

| 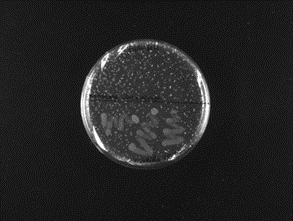  ***E.coli* BL21 (top half of plate) vs *P. putida* KT2440 (bottom half of plate)**  **M9 *n-*butanol silica gels** | 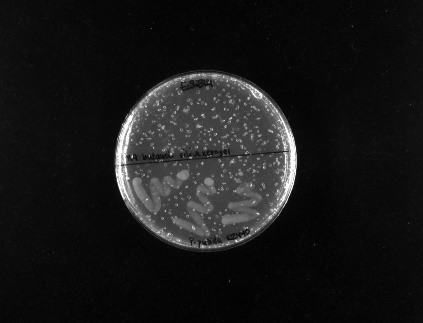 | 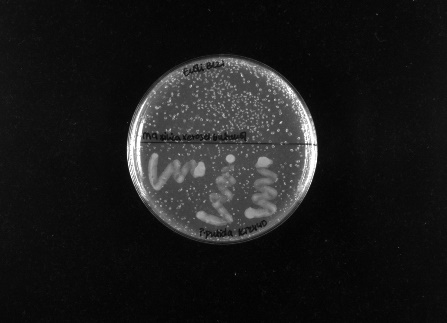 |
| --- | --- | --- |
| 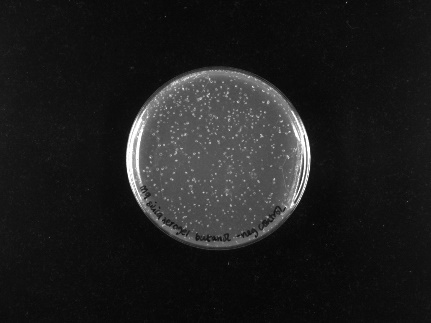 | 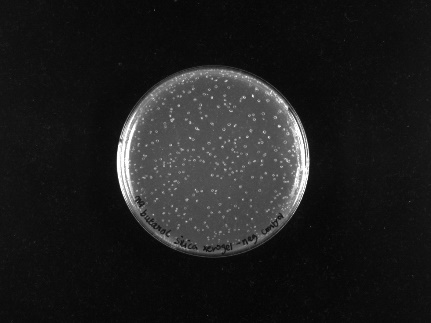  **Neg control – M9 *n-*butanol silica gels** | 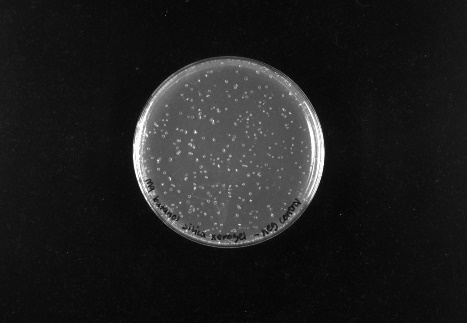 |

**Figure S8.** *E.coli* BL21 *vs P. putida* KT2440 grown on M9 *n*-butanol supplemented gels and their corresponding negative controls (gels without inoculum).

| 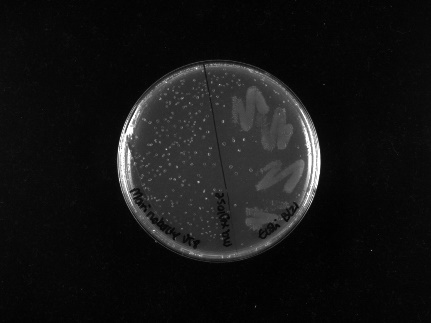 | 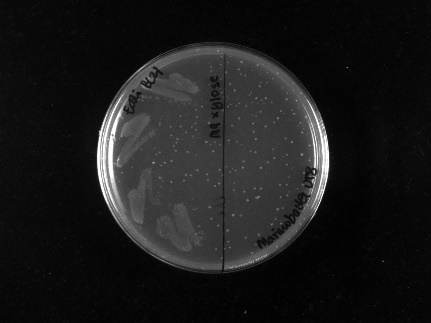 | 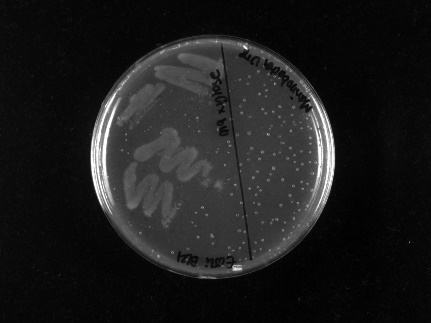  ***E.coli* BL21 (left half of plate) vs *M. nauticus VT8*** **(right half of plate)**  **M9 xylose silica gels** |
| --- | --- | --- |
| 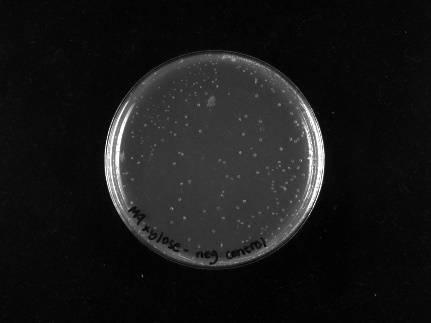 | 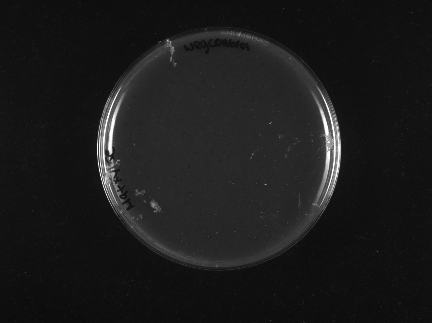 | 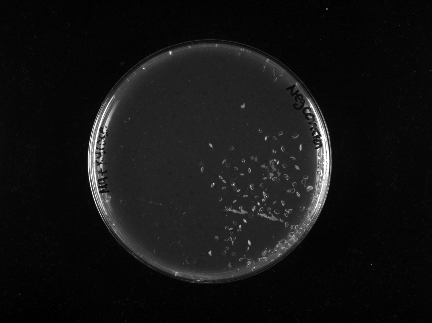 |

**Neg control – M9 xylose silica gels**

**Figure S9.** *E.coli* BL21 *vs M. nauticus* VT8 grown on M9 xylose supplemented gels and their corresponding negative controls (gels without inoculum)*.*
