# Supplementary material for: Silica hydrogels as a carbon-free solid media for the culture of diverse organisms
Source: FEMS Microbes. 2024 Dec 28;6:xtae035. doi: 10.1093/femsmc/xtae035 (PMC11737323; doi:10.1093/femsmc/xtae035)
Supplement: xtae035_Supplemental_Files [file L58_response_letter_v4.pdf › L58_response_letter_v4.pdf]

MANCHESTER 1824

The University of Manchester

Lu Shin Wong PhD MRPharmS
Senior Lecturer

Manchester Institute of Biotechnology
The University of Manchester
131 Princess Street
Manchester M1 7DN

Tel: +44 (0)161 529 4357
l.s.wong@manchester.ac.uk
www.chemistry.manchester.ac.uk
www.mib.ac.uk

Prof. Kathleen T. Scott
Editor-in-Chief
FEMS Microbes
Oxford University Press

22 November 2024

Dear Professor Scott,

Silica hydrogels as a carbon-free solid media for the culture of diverse organisms
Manuscript ID: FEMSMC-2024-041

Thank you for considering our manuscript. Please find attached the revised manuscript and supporting information documents. Our point-by-point response to the reviewers' comments is given below, together with the details of any changes in the revised manuscript (in blue text).

Reviewer #1:

1. *Have the authors investigated or have any comment on the sustainability of silica hydrogels? If these gels were to replace the use in whole, or in part of agar in microbiology would it be more or less sustainable either in its production or its disposal/ biological degradation etc*

Whilst the carbon footprint of silica hydrogels is likely to be higher than that of agar (the tetraethoxysilane is produced by metallurgical processes), agar production is itself unsustainable under current practices. Seaweed populations from which agar is derived are not readily cultivated, and overharvesting from natural sources have resulted in fluctuations in the supply in the recent past, with resultant global shortages. These issues were mentioned in the introductory section of the manuscript and have been the subject of other papers (see references 4 and 5 in the revised manuscript). The intention was therefore that these hydrogels could be used in scenarios where agar could not be procured due to shortages, and not as a wholesale replacement.

We have revised the text in the Conclusions section (Page 13 of the revised manuscript) to reflect this point:

"It is anticipated that these gels could serve as an alternative to agar in the event of supply shortages."

2. *The authors state that a use case for this gels would be the isolation of organisms that can degrade anthropogenic compounds without the confounding factor of the carbon in agar. Do the authors have any examples of specific compounds or families of compounds that would benefit from this approach?*

Yes, specific examples were already given in the manuscript. In the introduction section, to support this point, we cite a paper that systematically describes the ways in which agar can obscure growth tests for the utilization of potential carbon sources by the fungus *Aspergillus nidulans* (reference 6 in the revised manuscript). Another example is given in our Results and Discussion section, when we discuss specific experiments. Here, we reference a paper (reference 19) on the use of inorganic silica gels to control the source of carbon when culturing the highly oligotropic *Rhodococcus erythropolis*.

Reviewer #2:

1. *The use of silica gel as a solidifying agent is more extensive than noted in the current manuscript. Please evaluate whether at least the following literature should be touched on to provide a more thorough overview of topic, which goes back to the early 20th century:*

a) *Dietz A. S. and Yayanos A. A., Silica gel media for isolating and studying bacteria under hydrostatic pressure. DOI: 10.1128/aem.36.6.966-968.1978*

b) *Kingsbury J. M. and Barghoorn E. S., Silica gel as a microbiological medium: potentialities and a new method of preparation. DOI: 10.1128/am.2.1.5-8.1954*

c) *Bazylinski D. A. and Rosenberg F. A., Silica gel plates for culture of marine and nonmarine organisms. DOI: 10.1128/aem.39.4.934-934.1980*

It isn't really apparent that there is a significant advantage to what is proposed here over these. In particular, the Bazylinski and Rosenberg method seems potentially easier and capable of supporting colony growth.

We thank the reviewer for bringing these references to our attention. These papers have been evaluated and were found to use inorganic silicate salts for gel preparation, which are now known to offer less controllability of the gel properties in terms of mechanical properties (references 10 and 20 in the revised manuscript). Indeed, reference (a) had already alluded to this issue with the statement "*The gelled medium as prepared here is fragile compared with agar*", and reference (c) mentions "*The fact that the silica gel is somewhat less solid than the routinely used agar should be kept in mind during handling*". In our own hands we also found that these previously reported procedures were generally inconsistent, with even small variations during the preparation resulting in gels with very different properties.

Furthermore, gels produced from inorganic silicate precursors have a tendency towards syneresis (i.e. exudation of water) resulting in gels with a watery consistency on the surface. This effect means that microbial colonies grown on these materials diffuse and merge, making the isolation of single clones impossible (reference 19 in manuscript).

In contrast, the present procedure uses the controlled hydrolysis of the TEOS silicate precursor to consistently produce silica gels with agar-like mechanical properties. In practical terms they can be manipulated in exactly the same manner. We also demonstrate that our gels are applicable to a wide range of microbes (see Table 2).

To address the reviewer's comment, we have now revised the manuscript to include more information on the limitations of previous methods. We have also carried out another literature search and have identified papers as early as 1949. The revised text, on Page 3 of the revised manuscript, now reads:

"In microbiology, silica gels have been applied in a variety of experiments, including the encapsulation of microbes within the silicate matrix, with subsequent applications as biosensors or bioreactors[9-12]. Various formulation of silica hydrogels in streak plate format for microbiological purposes has been known since at least 1949[13-18]. These gel preparations were later used in environmental microbiology experiments to exclude the effect of any agarolytic activity[19]. However, previous efforts to produce silica gels using inorganic silicates have suffered from poor control of their mechanical properties (*i.e.* gel stiffness) which made them difficult to handle in practical microbiology[10, 20]. Furthermore, it has been found that these gels were difficult to use because of their watery consistency that resulted in the diffusion of the colonies (*i.e.* not possible to isolate individual ones) and any attempts at drying them resulted in gel cracking[19]"

New references 14-18 have also been added to the manuscript, including those mentioned by the reviewer.

2. *The method produces ethanol as a product of the gelling reaction. How complete is the conversion of TEOS during gelation? To test, please show the results of cultivating S. cerevisiae on YNB silica gel plates or P. putida KT2440 (mislabeled in Table 1?) with no*

carbon source, glucose, or 1% ethanol. If there is significant residual ethanol produced over time in the plates, both S. cerevisiae and P. putida should grow on the no carbon plate as they are capable on aerobic growth with ethanol as the sole carbon source. This is the last control I would want to see to really convince a reader that metabolizable carbon is not present in these plates.

As requested by the reviewer, to demonstrate that negligible amounts of ethanol (or any other carbon source) are present in the gels, two additional culture experiments were carried on gels containing: (i) M9 minimal media and inoculated with *P. putida* KT2440 or (ii) YNB media (without amino acids) and inoculated with *S. cerevisiae* 96.2, a strain that lacks nutritional deficiencies that inhibit growth in the absence of amino acids. In both cases, no colonies were observed, confirming that negligible (if any) ethanol was present in the prepared gels.

This result is now added to the revised manuscript:

"In order to test whether these silica hydrogels had any residual ethanol that could serve as a viable source of carbon, two further experiments were carried out whereby gels were prepared that did not contain any additional carbon source. Here, gels were prepared containing either M9 minimal medium and inoculated with *P. putida* KT2440; or containing YNB without amino acids and inoculated with *S. cerevisiae* 96.2, a strain that lacks nutritional deficiencies that inhibit growth in the absence of amino acids. In both cases no colonies were observed after overnight growth **(Figure S6 in SI)**."

Photos of these plates are now also included in the SI (Figure S6 in SI), and in the methodology section, descriptions are now included for the source of *S. cerevisiae* 96.2 and the procedure for the preparation of the YNB gels (Pages 4 and Table 1):

"*Saccharomyces cerevisiae* isolate 96.2 (*S. cerevisiae* 96.2) was provided courtesy of Eladio Barrio[21]."

S. cerevisiae 96.2	Yeast Nitrogen Base (YNB) media containing (in g L^{-1}): Yeast-Nitrogen-Base without amino acids and without ammonium sulphate (1.9), ammonium sulphate (5.0).	30 °C

The relevant reference 21 has also been added to the manuscript.

Other revisions:

A small number of formatting and typographical errors have also been corrected. Minor alterations to the wording have also been made to enable smoother reading and integration of the new content. These are all identified as blue text in the revised manuscript.

Thank you for your time and consideration. I look forward to your response.

Yours sincerely,

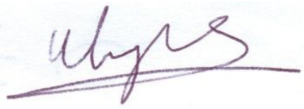

Lu Shin Wong
