# Supplementary material for: Silica hydrogels as a carbon-free solid media for the culture of diverse organisms
Source: FEMS Microbes. 2024 Dec 28;6:xtae035. doi: 10.1093/femsmc/xtae035 (PMC11737323; doi:10.1093/femsmc/xtae035)
Supplement: xtae035_Supplemental_Files [file xtae035_supplemental_files.zip › FEMSMC-2024-041.R1 supplementary data - reviews and author response.docx]

**Reviews of manuscript FEMSMC-2024-041**

**Reviewer 1**

**Recommendation: Accept**

Have the authors investigated or have any comment on the sustainability of silica hydrogels? If these gels were to replace the use in whole, or in part of agar in microbiology would it be more or less sustainable either in its production or its disposal/ biological degradation etc. The authors state that a use case for this gels would be the isolation of organisms that can degrade anthropogenic compounds without the confounding factor of the carbon in agar. Do the authors have any examples of specific compounds or families of compounds that would benefit from this approach?

**Reviewer 2: Thomas Hanson, University of Delaware**

**Recommendation: Minor Revision**

The manuscript presents a different method of preparing silica gel plates for microbial cultivation. The data are reasonable, but I have two significant concerns.

The use of silica gel as a solidifying agent is more extensive than noted in the current manuscript. Please evaluate whether at least the following literature should be touched on to provide a more thorough overview of topic, which goes back to the early 20th century:

-Dietz AS, Yayanos AA. 1978. Silica gel media for isolating and studying bacteria under hydrostatic pressure. Appl Environ Microbiol https://doi.org/10.1128/aem.36.6.966-968.1978

-KINGSBURY JM, BARGHOORN ES. Silica gel as a microbiological medium: potentialities and a new method of preparation. Appl Microbiol. 1954 Jan;2(1):5-8. doi: 10.1128/am.2.1.5-8.1954

-Bazylinski DA, Rosenberg FA. Silica gel plates for culture of marine and nonmarine organisms. Appl Environ Microbiol. 1980 Apr;39(4):934. doi: 10.1128/aem.39.4.934-934.1980.

It isn't really apparent that there is a significant advantage to what is proposed here over these. In particular, the Bazylinski and Rosenberg method seems potentially easier and capable of supporting colony growth.

The method produces ethanol as a product of the gelling reaction. How complete is the conversion of TEOS during gelation? To test, please show the results of cultivating S. cerevisiae on YNB silica gel plates or P. putida KT2440 (mislabeled in Table 1?) with no carbon source, glucose, or 1% ethanol. If there is significant residual ethanol produced over time in the plates, both S. cerevisiae and P. putida should grow on the no carbon plate as they are capable on aerobic growth with ethanol as the sole carbon source. This is the last control I would want to see to really convince a reader that metabolizable carbon is not present in these plates.

**Editor Decision: Minor Revision**

**Author's Response to Decision Letter for (FEMSMC-2024-041)**

RESPONSE LETTER IS INCLUDED AS SUPPLEMENTARY FILE FOR REVIEW TITLED ‘L58_response_letter_v4.pdf (v1.0)’

**Reviews of manuscript FEMSMC-2024-041.r1**

**Reviewer 1**

**Recommendation: Accept**

Happy with changes and comments made in response to initial review

**Reviewer 2: Thomas Hanson, University of Delaware**

**Recommendation: Accept**

Thank you for the clear responses**.**

**Editor Decision: Accept**
